# Primary stability of multi-hole cups compared to plate osteosynthesis in osteoporotic anterior column and posterior hemi-transverse acetabular fractures—A biomechanical comparison

**DOI:** 10.1371/journal.pone.0270866

**Published:** 2022-07-27

**Authors:** Andreas Höch, Rebekka Reise, Philipp Pieroh, Christoph-Eckhard Heyde, Johannes Karl Maria Fakler, Stefan Schleifenbaum

**Affiliations:** 1 Department of Orthopaedic Surgery, Traumatology and Plastic Surgery, University of Leipzig Medical Center, Leipzig, Germany; 2 ZESBO–Center for Research on Musculoskeletal System, Leipzig University, Leipzig, Germany; University Hospital Zurich, SWITZERLAND

## Abstract

**Introduction:**

Acetabular fractures pose high demands on the surgeon and in the case of osteosynthetic treatment, anatomical reconstruction has the highest priority to achieve a good outcome. However, especially in older patients with poor bone quality, even anatomical reconstruction is no guarantee for a good clinical outcome and may nevertheless end in early osteoarthritis. Primary arthroplasty therefore has an increasing importance in the treatment of these patients. The aim of this study was to biomechanically compare fracture gap displacement and failure load as an assessment measure of the primary stability of conventional plate osteosynthesis with the treatment using a sole multi-hole cup for acetabular fractures.

**Methods:**

Six hemi-pelvises each with anterior column and posterior hemi-transverse (ACPHT) fracture were treated with either plate osteosynthesis or a multi-hole cup. The tests were carried out in a standardised test set-up with cyclic loading in various stages between 150 N and 2500 N. The fracture gap displacement was recorded with optical 3D measuring and the failure load was determined after the cyclic measurement.

**Results:**

With increasing force, the fracture gap displacement increased in both procedures. In each group there was one treatment which failed at the cyclic loading test and a bone fragment was broken out. The primary stability in arthroplasty was comparable to that of the standard osteosynthesis.

**Conclusions:**

The results found seem promising that the primary arthroplasty with a sole multi-hole cup and corresponding screw fixation achieves an initial stability comparable to osteosynthesis for typical ACPHT fractures. However, further clinical studies are needed to prove that the cups heal solidly into the bone.

## Introduction

Acetabular fractures pose a great challenge to the surgeon and require anatomical reduction for a good clinical outcome [1, 2]. Currently, the proportion of acetabular fractures in older patients with reduced bone quality is increasing in all industrial nations [3, 4]. Osteomalacia or manifest osteoporosis is usually present and leads to typically differences in fracture classification and the morphology of fractures with especially central impressions and bone defects in the area of “dome impaction” [3, 5, 6]. Even with excellent anatomically reduction these aspects can lead to an early failure of the osteosynthesis and consequently lead to a rapidly progressing osteoarthritis of the hip joint [7–10].

Therefore, the primary arthroplasty of acetabular fractures in elderly is increasingly discussed to avoid unnecessary secondary interventions [6, 9, 11, 12].

But so far, it should be critically considered if the sole arthroplasty is stable enough or if an additional plate osteosynthesis is required. Noteworthy, in the majority of cases two surgical approaches are needed for arthroplasty with additional plate osteosynthesis probably increasing the risk for surgical site infection [13, 14]. For primary arthroplasty alone, more complex revision cups with tabs, augments or cones are usually used [15, 16].

However, it remains unclear if "standard" cups with various possibilities of screw connection may provide enough stabiltiy for osteointegration within the osteoporotic bone.

Here, we compared plate osteosynthesis to multi-hole acetabular cup implementation treating anterior column and posterior hemi-transverse (ACPHT) acetabular fracture in terms of fracture gap displacement and failure load as an assessment measure of primary stability.

## Materials and methods

### Sample preparation

Twelve right hemi-pelvis bones with foam cancellous core (Sawbones, large, 4^th^ generation composite, Pacific Research Laboratories, Inc., Vashon, WA, USA) and the same batch number were used in this study. They have an advantage of standardization in construct behavior and ensure the homogeneity of the samples. Therefore, a number of 6 per group is sufficient. The samples were embedded in casting resin (mixture of the basic materials RenCast® FC 52/53 isocyanate, FC 53 polyol (Huntsman Advanced Materials, Basel, Switzerland) and aluminium hydroxide (Füller DT 082, Gößl + Pfaff GmbH, Karlskron/Brautlach, Germany)) at the posterior iliac spine with an embedding construction and a template. The embedding served as a fixation point for the testing machine so that the load applied by the machine happened to be anatomically correct and reproducible. The corresponding orientation of hip bones and the force vector of the maximum load during walking are based on literature and the publication by Bergmann et al. [17].

A reproducible osteotomy using a template in terms of an anterior column and posterior hemi-transverse (ACPHT) acetabular fracture according to Letournel was carried out on all specimens [2]. The samples were divided into two experimental groups of six artificial bones each. Furthermore they were prepared and treated by the same experienced pelvic surgeon.

**Fig 1:**
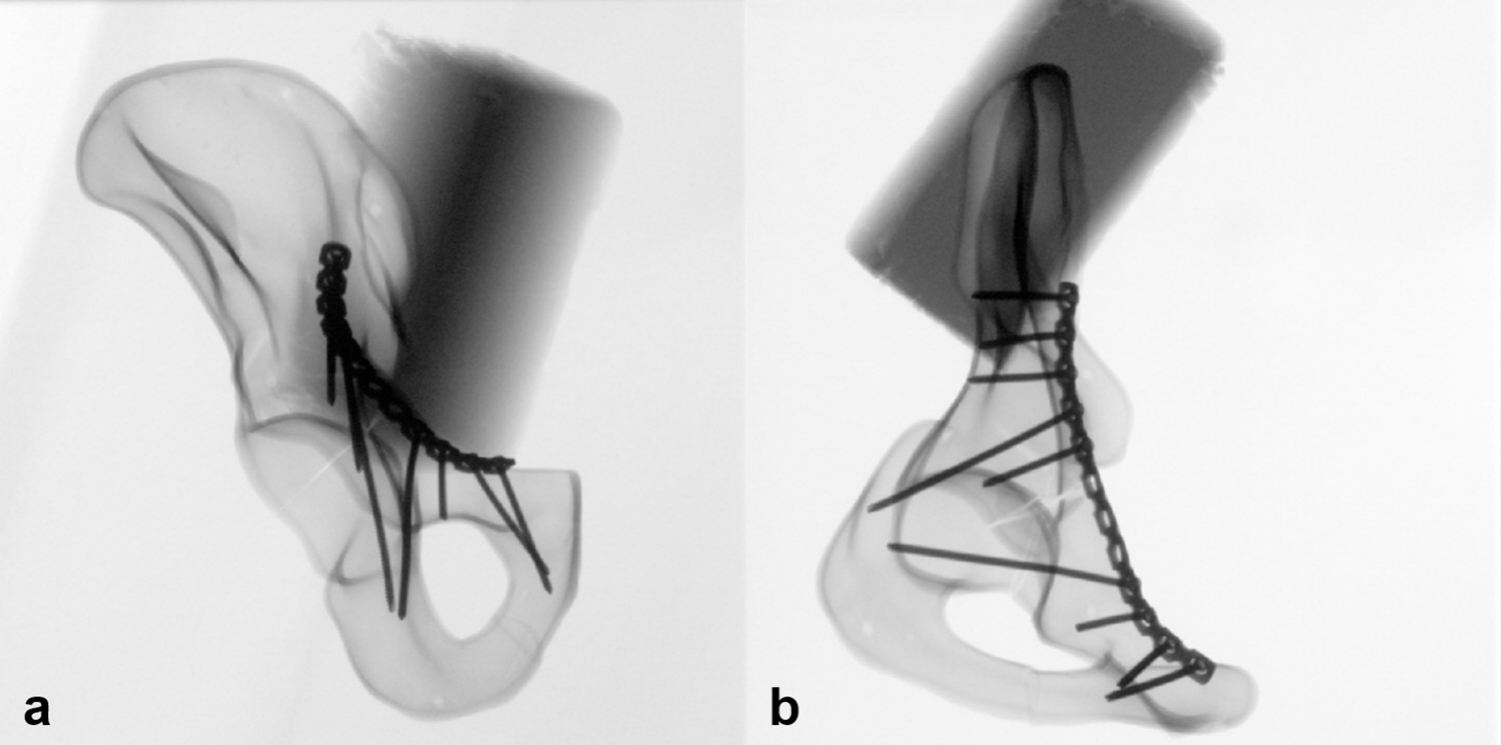
X-ray imaging of the pelvis model with plate osteosynthesis. **a** shows the a.p.- view and **b** the obturator- view of the hemi-pelvis.

In group 1, the acetabular fracture was treated using a 3.5 mm 12-hole reconstruction plate (DePuy Synthes Comp., Zuchwil, Switzerland). For reconstruction, the fractured pelvis was temporarily fixed with K-wires and then definitively treated with plate osteosynthesis. The screw position was identical in all pelvises (Fig 1A and 1B).

**Fig 2:**
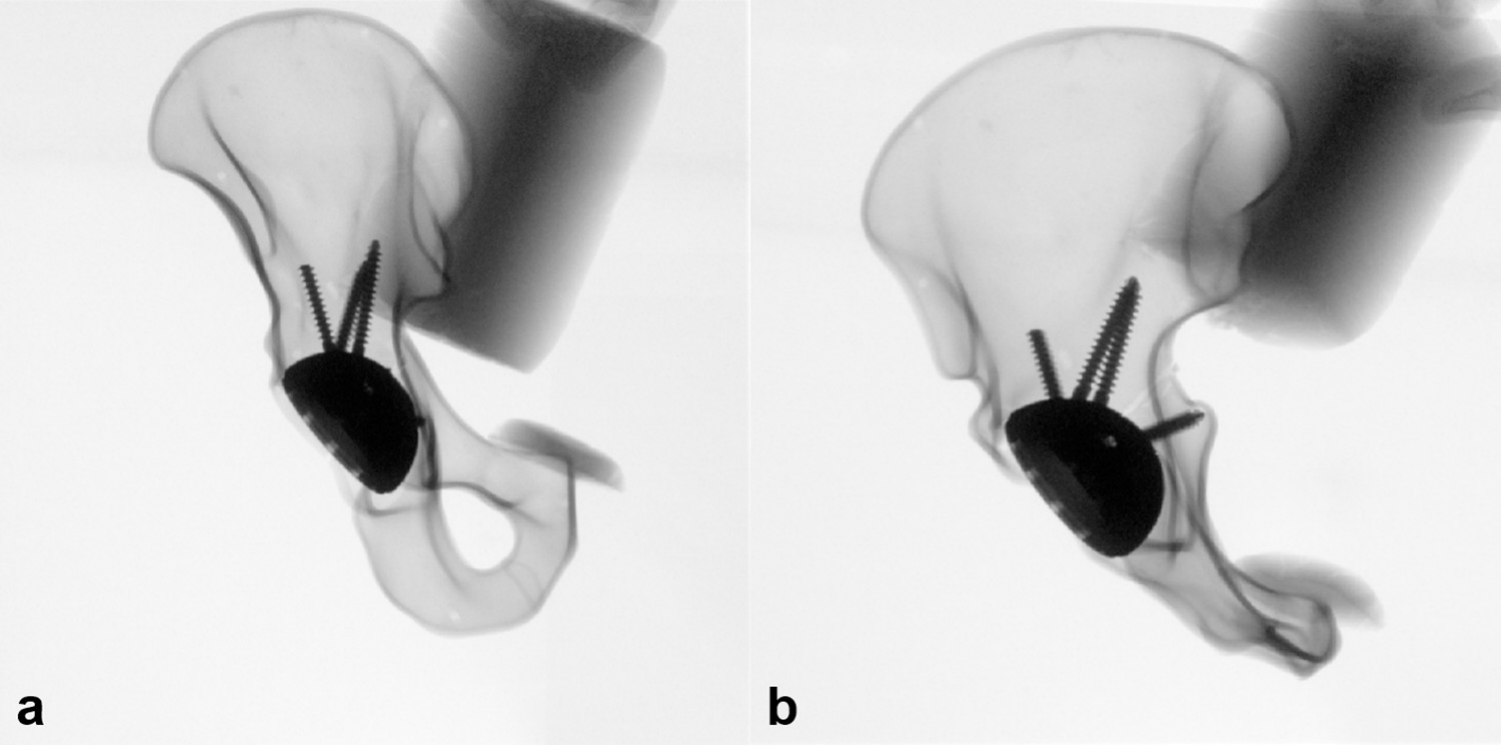
X-ray imaging of the pelvis model with arthroplasty. **a** shows the a.p.- view and **b** the ala- view of the hemi-pelvis.

Group 2 was treated with a 58 mm multi-hole cup (Pinnacle Multihole, DePuy Synthes Comp., Zuchwil, Switzerland). After temporary fixation, the acetabulum was reamed and the multi-hole cup was implanted and fixed with four screws according to the possibilities of a directlateral approach to the hip [18]. Identical screw positioning and screw length was used for all pelvises (Fig 2A and 2B).

### Set up

**Fig 3:**
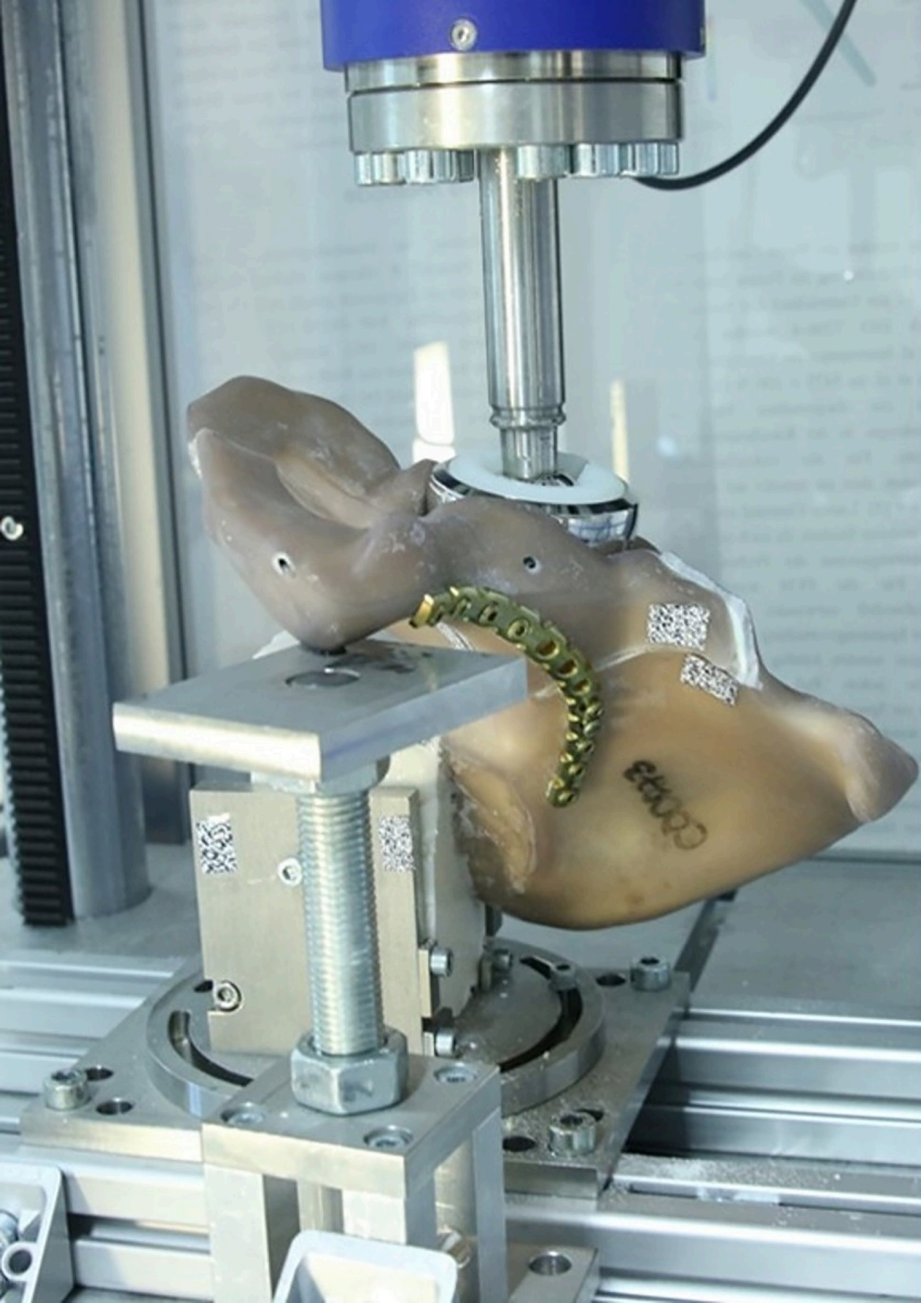
Test set-up clamped in the testing machine. The acetabulum was aligned in a manner that a physiological force introduction was realized according to Bergmann et al.

The prepared artificial hip bones were fixed in the uniaxial test machine (DYNA-MESS Prüfsysteme GmbH, Stolberg, Germany) with a self-developed test rig. The hemi-pelvis was fixed in place with a rotating plate that aligns the embedded posterior iliac spine according to the desired fracture examination. After the correct adjustment of the specimen orientation, the plate was attached with screws. The cast resin was fixed with additional plates and screws. The symphysis respectively the embedded part of the symphysis was supported on a height-adjustable bearing surface. The surface was lubricated. This set up ensures comparable starting situations. In addition, the axial force introduction of the load according to Bergmann et al. is implemented with this design (Fig 3) [17].

In group 1 a bipolar head was used for force application and in group 2 a 32 mm ceramic head with matching polyethylene inlay was used for the examination of the multi-hole cups.

### Cyclic load test

Many studies advocate early full loading to counteract physical and mental degradation and most elderly patients are unable to perform a postoperative partial weight-bearing therapy [19–23]. Therefore the cyclic load test was carried out in a range between 150–2500 N and thus simulates the load when walking for patients with average body weight [24]. The examination was divided in five load levels with 500 cycles each and the sinusoidal load was applied at a frequency of 1 Hz. The specimens are preloaded with 150 N to create standardized initial conditions and to avoid settling effects. The values of the force applied, and the distance traveled were continuously recorded. The test was stopped when the respective treatment failed, bone fragments broke away or the force dropped by 300 N due to a large fracture displacement.

### Optical 3D measurement

**Fig 4:**
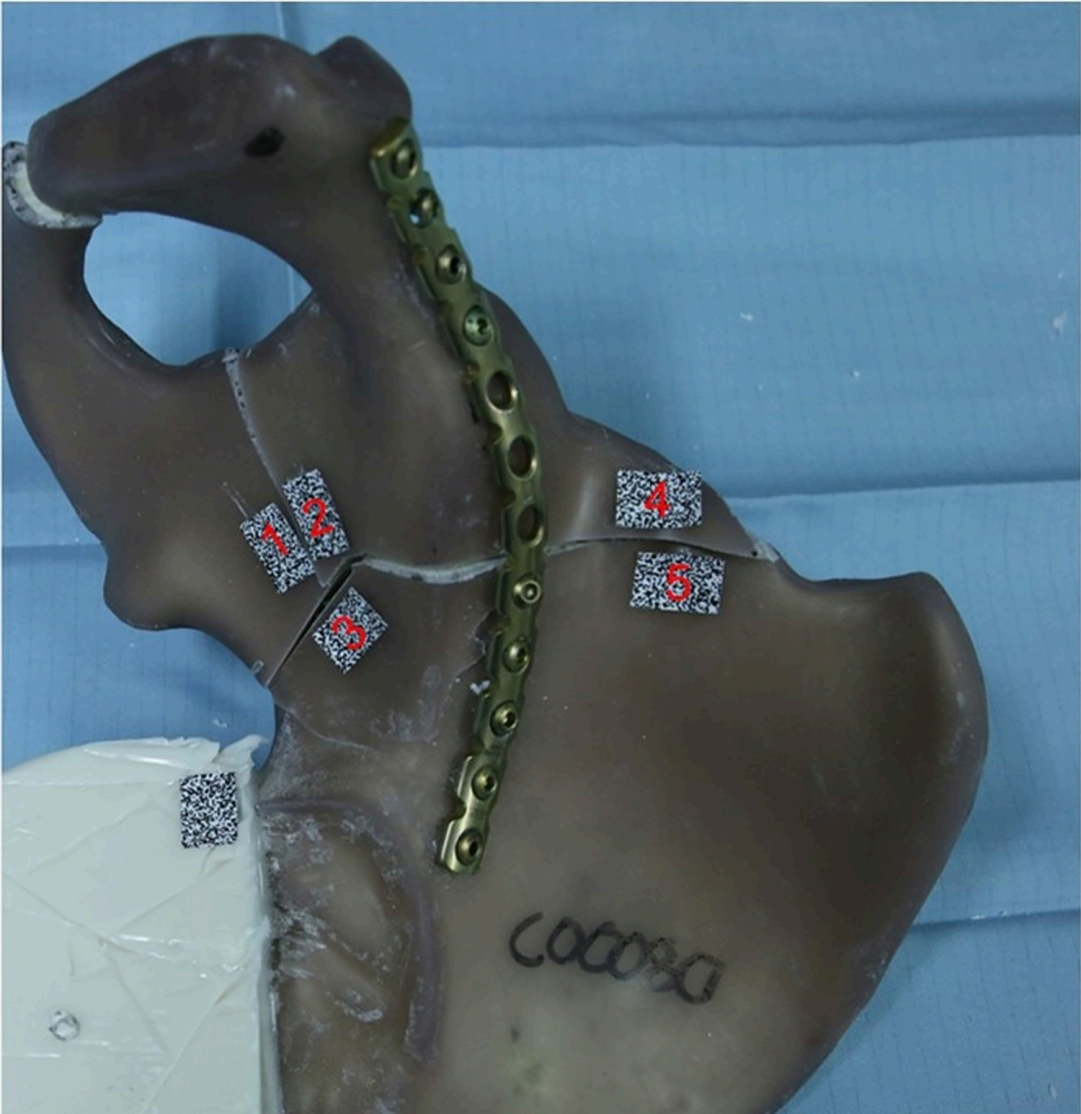
Shows the standardized placement of the spackle patterns. Four different fracture gap displacements were measured. The optical measurement was performed between spackle pattern 1/2, 1/3, 2/3 and 4/5 (1 = os ischii; 2 and 4 = os pubis; 3 and 5 = os ilium).

Several markers with speckle patterns were placed on each fracture fragment to measure the displacement in the fracture gaps (Fig 4). The fracture displacements were observed and recorded with a 3D measuring system (LIMESS Messtechnik & Software GmbH, Krefeld, Germany), which has an accuracy of 0.01 pixel or 1 μm in a three-dimensional movement analysis [25]. It was linked to the testing machine and allowed images to be stored after the preload and at each load level.

The analysis of the displacement between marker 1 and 2, 1 and 3, 2 and 3, and 4 and 5 was determined using the correlation software Istra 4D (Dantec Dynamics A/S, Skovlunde, Denmark) and a self-developed MATLAB routine (MATLAB (R2020b), MathWorks, Natick, USA).

### Failure test

The failure test was performed using a path-controlled test at a speed of 0.2 mm/s. In case bone fragments or the corresponding implant failed out or the force exerted by the testing machine dropped by 300 N, the test was stopped and the maximum achieved force was documented.

### Statistical evaluation

Statistics were calculated using SPSS 24.0 for Windows® (SPSS inc., Chicago, IL, USA). To verify the effect of fracture displacement on osteosynthesis and arthroplasty, the data was first tested for normal distribution using the Shapiro-Wilk test and then tested on significance with the non-parametric Mann-Whitney-U test, because the samples are not normally distributed and unconnected. All statistical procedures were performed with a significance level of α = 0.05.

## Results

In each of the two groups, one sample failed at the highest cyclic load level of 2500 N. The specimen of the osteosynthesis group failed after 2300 cycles and the other one of the multi-hole cup treatment failed at cycle 2010. In 2/6 samples of group 1 the force caused minor fractures at the acetabular rim.

### Fracture gap displacement

The maximum fracture gap displacement increased in both groups with increasing load in all fracture gaps examined.

**Fig 5:**
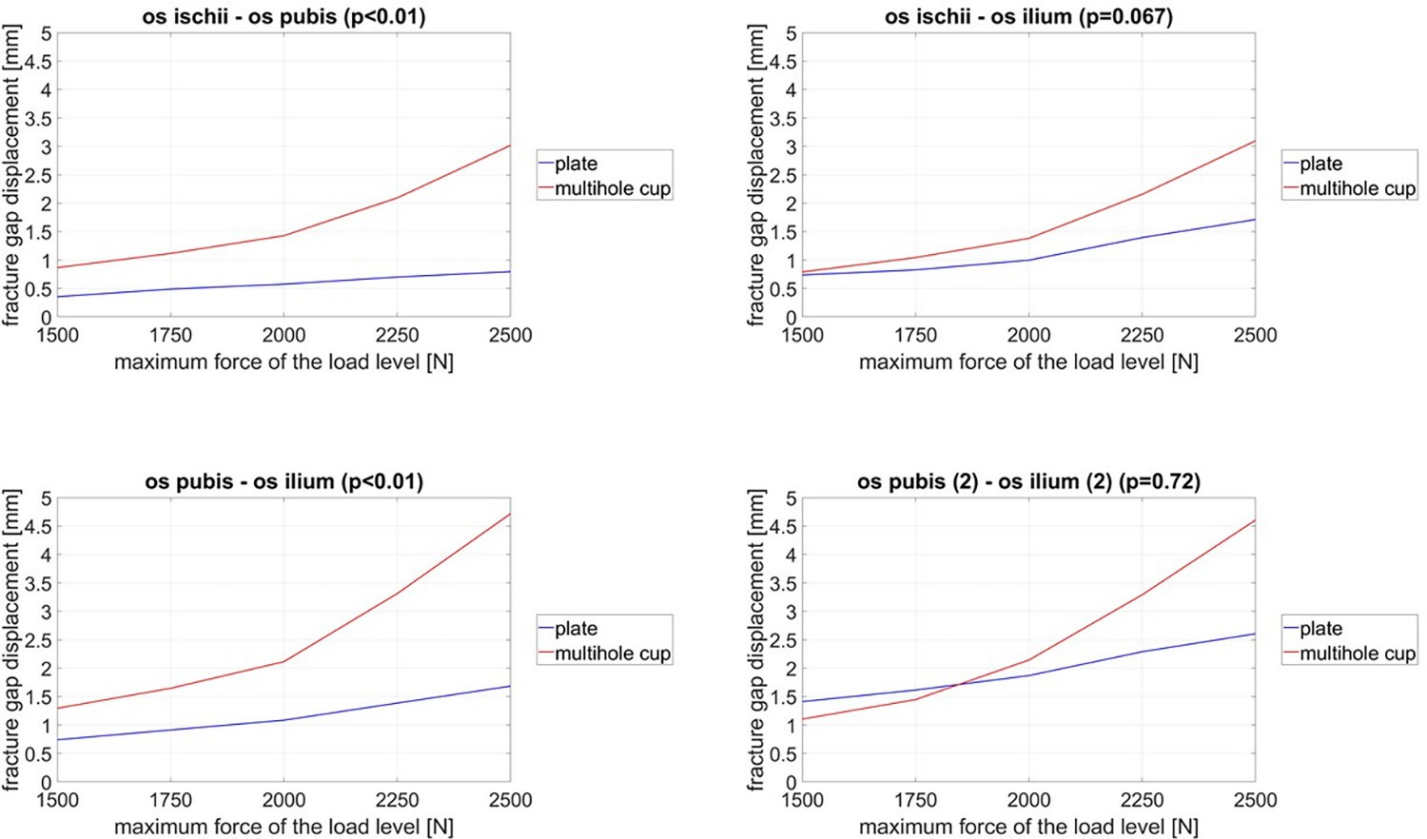
Graphs show the fracture gap displacement between the different measuring points as a function of the applied forces during cyclic loading.

The comparison of the two groups demonstrates that the average increase of the maximum displacement between marker 1 and marker 2 and between marker 2 and 3 is smaller in experimental group 1 than in group 2 (p<0.01) (Fig 5).

The displacement in the fracture gap between marker 1 and marker 3 of the two experimental groups was equal (p = 0.067). In contrast, the average fracture gap displacement between marker 4 and marker 5 was higher in osteosynthesis at the first two loading stages and in the further course the displacement was higher in the multi-hole cups group (p = 0.72).

### Failure test

**Fig 6:**
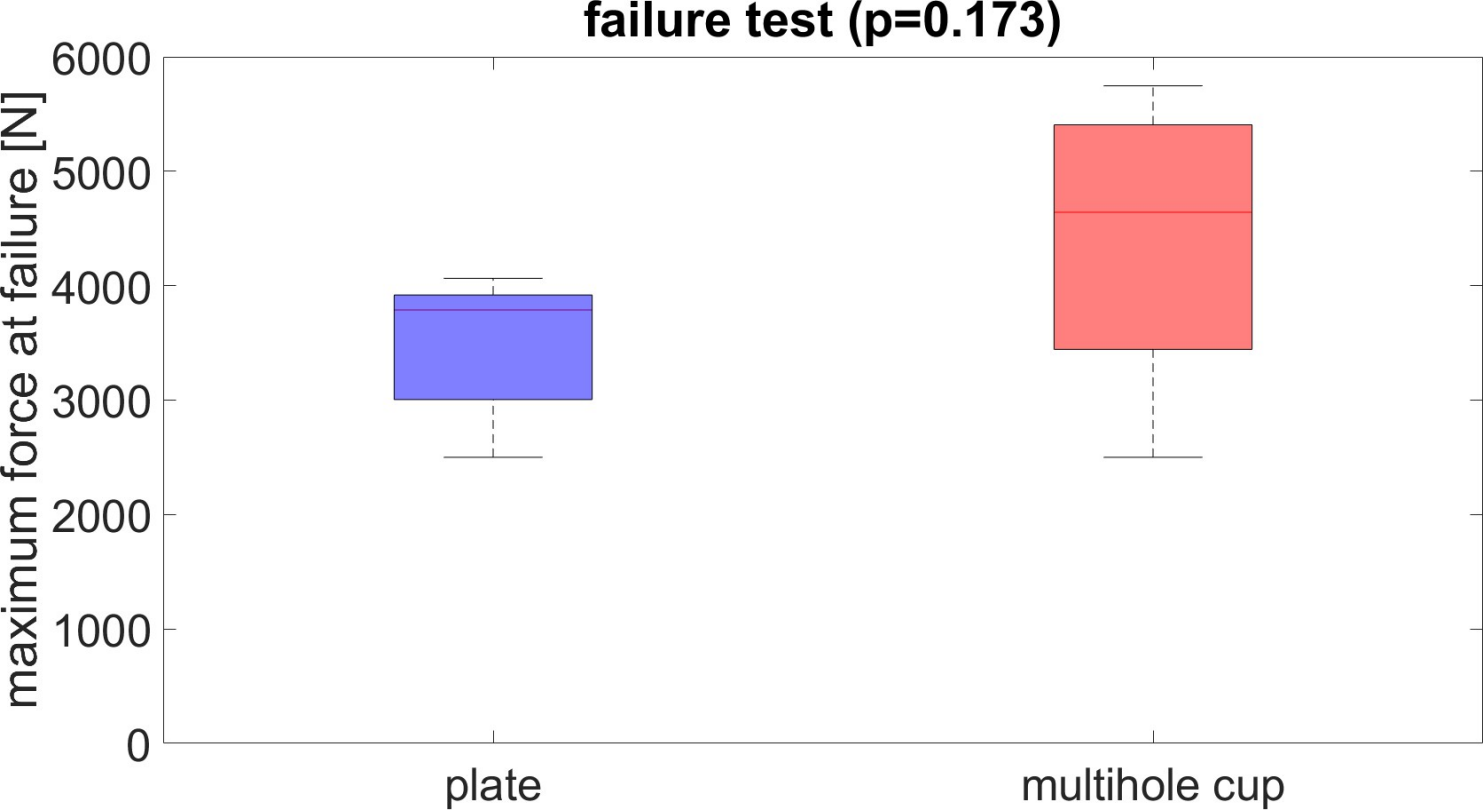
Boxplots compare the maximum failure force of the osteosynthesis and the arthroplasty without significant difference (p = 0.173).

The maximum load at failure testing was comparable for the multi-hole cup (4396.6 N ± 1130.8) and for osteosynthesis (3510.7 N ± 564.1) (p = 0.173) (Fig 6).

## Discussion

The present biomechanical study compares the primary stability of a classical treatment of ACPHT acetabular fractures using plate osteosynthesis with the primary arthroplasty using a multi-hole cup.

For our study, a multi-hole cup was deliberately chosen that can be implanted via a direct lateral access, including the screw positions selected in our setup [18]. Compared to more complex revision systems for hip arthroplasty, we assume that access-related complications, especially infection rates, can be reduced using a common multi-hole cup [21]. However, there is not much literature on this assumption.

We decided to test the ACPHT fracture as this is one of the most common acetabular fracture in old patients following low energy trauma [22]. However, we are aware that the sole arthroplasty of e.g. 2-column fractures is usually not possible and the decision on therapy must be very individually adapted to the respective patient and the type of fracture. In the cases a combination of plate osteosynthesis and arthroplasty can be necessary [23]. On the other hand, there is the relevant problem of cup loosening, which can lead to elaboraterevision operations with socket reconstruction. This is a well-known and relevant problem in biomechanical testing, since biological migration of the implants and bone healing cannot be depicted.

In contrast to the present study it must be emphasized that not all studies used an experimental set-up with an anatomical force vector in the sense of a single-legged stand [11, 24, 25]. Furthermore, most authors have not tested a full weight bearing or defined it very diversely, which makes it impossible to compare the results [25–27]. In our opinion, however, testing with full weight bearing makes sense, since older patients are rarely able to implement partial weight bearing and therefore the possible full weight bearing should always be the aim of treatment in elderly patients [10, 28]. There is only one study known to us that used a similar experimental setup and investigated the primary stability of arthroplasty of acetabular fractures with revision cups alone. However, in contrast to our study, no failure test was performed [11]. Compared to the previous studies, our test setup allows both test variants, a failure test and a cyclic loading test with the anatomically correct force direction while walking.

In addition to the generally known limitations of biomechanical testing, the simulation of the one-legged stance with maximum force while walking must be viewed critically. There is always the same force application considered without any change of the force vectors, as they occur e.g. when sitting.

Furthermore, the two fractures that occurred at the acetabular rim may indicate that there was a difference in the force application between the bipolar-head on the native joint and the ceramic head that applies the load through the implanted cup.

As expected and shown by other biomechanical studies, fracture gap motion increased under increasing load in all hemi-pelvises independent of the treatment.

The results reveal an advantage of plate osteosynthesis for the fracture gap displacement. Nevertheless, the primary arthroplasty with multi-hole cups seems to be at least equal in the comparison of the maximum failure force. The presented results obtained comparable values of failure load as Busuttil et al. published for the stability of percutaneous screw fixation and plate osteosynthesis in ACPHT acetabular fractures [24]. Although it is known that loosening of total hip replacement can lead to complaints in patients, this was only indirectly evaluated by fracture gap displacement.

Nevertheless, we consider the present results to be promising. The aim of treatment for elderly patients with acetabular fracture should always be the rapid implementation of full weight bearing and the minimization of surgical approach related morbidity.

This may be achieved by primary arthroplasty and should be improved by technical advancements, if possible via an isolated surgical approach. Looking ahead, implant developments with different screw options specifically adapted for fracture treatment and bone-integrating surfaces are certainly useful in this context.

## Conclusion

Despite the limitations, the present results seem promising that primary THA without additional osteosynthesis is an option of therapy if good anchorage is available. Primary stability seems comparable in the treatment of ACPHT fractures. However, clinical studies would have to be connected to verify this in terms of functional outcome and pain. The developed test setup delivered reliable results. Due to the setup, experiments with human donor preparations could be carried out.

## Supporting information

S1 Table Average value of the maximum fracture gap displacement and standard deviations. (XLSX)

S2 Table Mean maximum fracture gap displacement of five specimens per group. (XLSX)

S3 Table Mean value and standard deviation of the maximum load at failure test (p = 0.173 (Mann-Whitney-U-test)). (XLSX)
